# Recurrent Atrial Flutter Requiring Multiple Cardioversions in a Preterm Infant

**DOI:** 10.1016/j.jaccas.2021.02.006

**Published:** 2021-04-21

**Authors:** Jesus C. Jaile, Manoj K. Gupta

**Affiliations:** a Department of Pediatrics, Harlem Hospital Center, Affiliate of Columbia University, New York, New York, USA; b Division of Pediatric Cardiology, Children’s Hospital at Montefiore, Bronx, New York, USA

**Keywords:** arrhythmia, atrial flutter, cardioversion, preterm infant, recurrent, AFL, atrial flutter, AV, atrioventricular, DOL, day of life, ECG, electrocardiogram, NICU, neonatal intensive care unit

## Abstract

We describe the first case of atrial flutter requiring multiple cardioversions in a preterm infant. Direct current cardioversion is one of the best-understood treatment options, with a first-time success rate higher than 96%. The electrocardiograms provided reveal a second run of atrial flutter occurring after successful cardioversion. (**Level of Difficulty: Intermediate.**)

## Central Illustration

**Figure:**
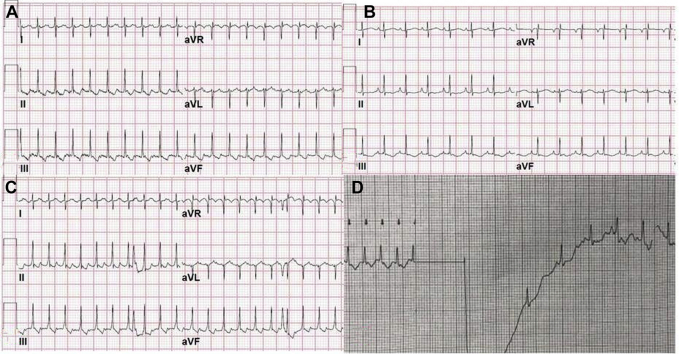

**Figure 1:**
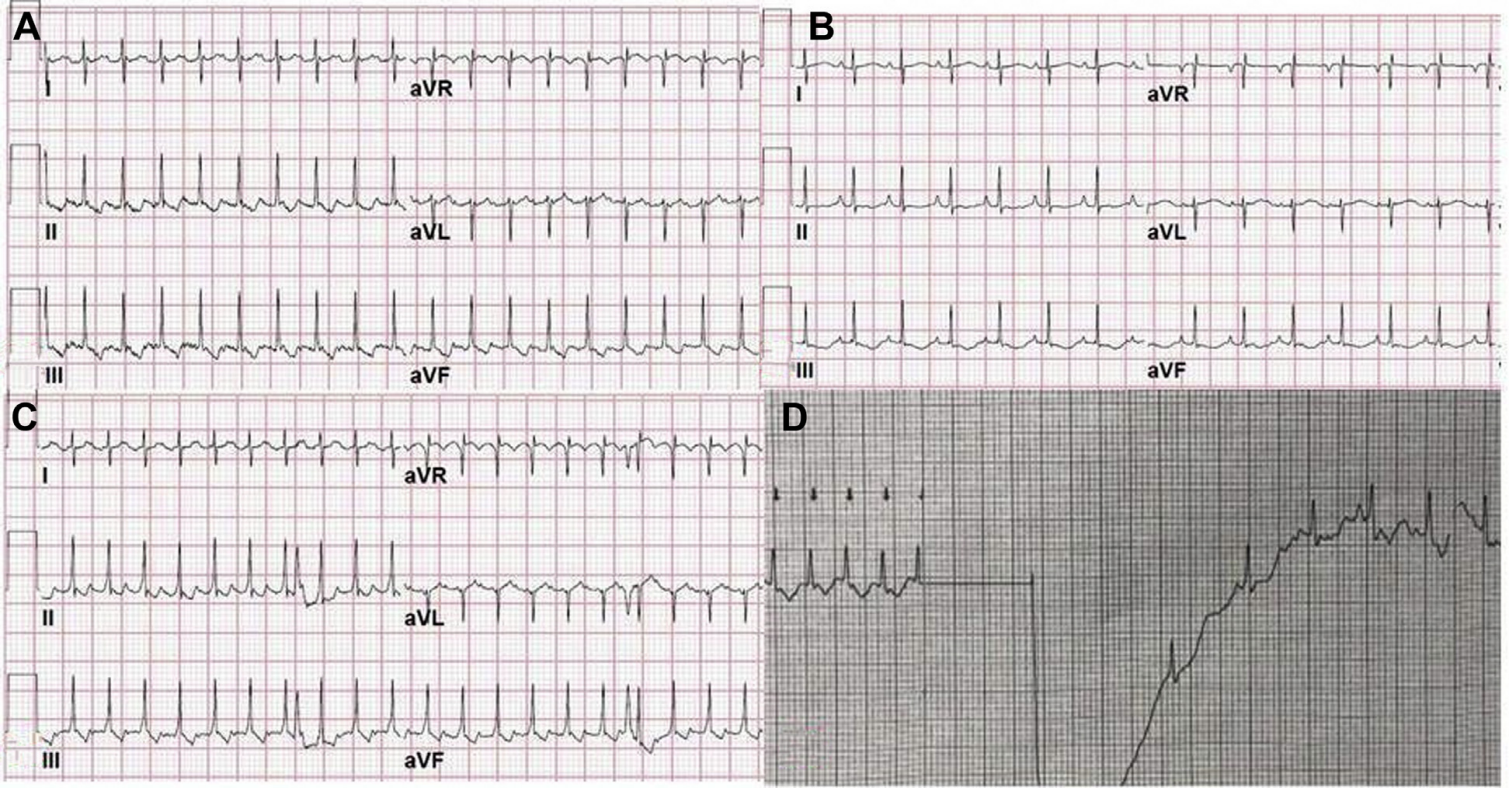
Electrocardiograms Showing Atrial Flutter and Sinus Rhythm Post Cardioversion **(A)** Electrocardiogram obtained on day of life 3 showing atrial flutter with a heart rate of 250 beats/min and a 2:1 atrioventricular block. **(B)** Electrocardiogram obtained after the first cardioversion showing normal sinus rhythm, heart rate 150 beats/min. **(C)** Second occurrence of atrial flutter, heart rate 250 beats/min, 28 h after the first episode. **(D)** Rhythm strip obtained from the defibrillator during the second cardioversion.

A 26 weeks’ gestation, a 1,035-g, preterm male infant was born by spontaneous vaginal delivery with Apgar (appearance, pulse, grimace, activity, respiration) scores of 7 and 7. The maternal prenatal history was unremarkable in terms of substance abuse, prenatal laboratory test results, and family history of cardiac disease. The history was pertinent for limited care secondary to delay in recognition of pregnancy resulting in no anatomy scans or antenatal ultrasounds. At birth, the baby received surfactant through an endotracheal tube, was extubated, was placed on continuous positive airway pressure, and was managed in the neonatal intensive care unit (NICU). Umbilical venous and arterial catheters were placed after birth and were confirmed to be in good position on radiographs, with the venous line tip at the right atrial–inferior vena cava junction. On day of life (DOL) 2, an echocardiogram was obtained for a heart murmur, which showed a small to moderate size patent ductus arteriosus, a patent foramen ovale, and borderline left ventricular systolic function at 54%. On DOL 3, he was started on caffeine for apnea of prematurity at a dose of 5 mg/kg/day. Results of blood culture, complete blood count, inflammatory markers, and electrolyte studies on the infant, including renal and hepatic studies, were unremarkable. During his NICU stay, vital signs and hemodynamic values remained stable and within normal limits for his age. There were no episodes of apnea, bradycardia, or desaturation. On DOL 4, at 87 h of life, cardiac monitoring showed a heart rate of 250 beats/min, and the cardiorespiratory monitor ECG pattern was suggestive of supraventricular tachycardia. ECG leads were placed, and an adenosine bolus was pushed according to protocol to break the tachycardia. This resulted in depiction of atrial flutter (AFL) waves with atrioventricular (AV) block, and then the rhythm strip showed AFL with 2:1 AV block (Figure 1A). Synchronized cardioversion was performed at 1 Joule/kg, and the rhythm converted to sinus at 150 beats/min (Figure 1B). There were no flutter waves, ectopic beats, or premature beats at baseline, and there was no re-initiation of the arrhythmia immediately following cardioversion. At 28 h following the cardioversion, the cardiorespiratory monitor showed tachycardia at 250 beats/min. Repeat ECG confirmed a second run of AFL with 2:1 AV block (Figure 1C). The patient underwent successful cardioversion with 1 Joule/kg (Figure 1D). The patient then remained in sinus rhythm for the whole duration of the NICU stay and was not started on any antiarrhythmic medications. Failure to convert AFL with cardioversion is highly suggestive of an autonomic mechanism or underlying medical condition. Our patient had normal results of thyroid function tests, newborn screening, and electrolyte tests during those 2 days.

## DISCUSSION

There are only 2 reported cases of recurrent AFL following cardioversion in the newborn period (1,2) and no known cases described in a preterm infant. Cardioversion of AFL regardless of gestational age has a first-time success rate higher than 96% (3). We report a unique case of AFL requiring multiple cardioversions in a preterm infant.

## Funding Support and Author Disclosures

The authors have reported that they have no relationships relevant to the contents of this paper to disclose.
